# Considerations on the Reciprocal Influence of the Physical Organization and Mental Manifestations

**Published:** 1855-11

**Authors:** A. O. Kellogg

**Affiliations:** Port Hope, Canada West


*Considerations on the Reciprocal Influence of the Physical Organization and Mental Manifestations.* By A. O. KELLOGG, M. D., Port Hope, Canada West.

On the intimate Relation of Epidemic Physical Disease, Popular Delusion, and Insanity—particularly as illustrated by the Epidemics of the Middle Ages, and the Epidemic and Popular Superstitions of our own Times.

There is nothing more evident than the fact, that it is impossible to study rightly and advantageously the diseases of the mind, without acquainting ourselves thoroughly, in the first place, with physical disease. Neither, on the other hand, can we understand or treat physical diseases successfully, without thoroughly understanding and appreciating the extraordinary influence of the mind upon all the bodily functions. In our last article, the advantage to be derived from a due consideration of this influence was pointed out, in some remarks respecting the successful treatment of dyspepsia by charlatans.

It is extremely interesting to observe, in reading the history of the epidemic diseases which from time to time have swept over the earth, that these have been accompanied or shortly succeeded by certain morbid, moral and intellectual manifestations; and between these physical, moral and mental ills, I am led to believe, there was a mysterious connection, which has not as yet been thoroughly traced out. Brains, previously weakened by these physical plagues, were left in a fit state to be morbidly impressed and led astray by the first moral or mental delusions which presented themselves to the multitude.

In the writings of the learned German, Dr. Hecher, from which we shall draw largely for illustration, much may be found to show the truth of this; and, among the diseases referred to, the *black death*, or plague, holds a prominent place This most appalling of maladies began in China, first spread over Asia, and traveling eastwards, like all great epidemics, entered Europe in the year 1348. This spread from the south to the north of Europe, occupying nearly three years in its passage. In the second year it had reached Sweden, in the third invaded Russia.

The civil and political condition of Europe, at the time of this invasion, rendered people peculiarly susceptible of its blighting influences.—War raged with demoniacal fury; robbery and violence shut up the peaceful citizens in walled cities and unwholesome habitations, overlooking filthy streets; within were pestilence and famine, without war, rapine and violence; the evil passions of humanity were left uncontrolled; religion was only another name for the darkest and grossest superstition and ignorance; and upon men weighed down by these accumulated evils came this dire pestilence, which in China alone was said to have destroyed thirteen millions—in Cairo fifteen thousand in a single day;* and it was said that vessels whose crews had perished were drifted about in the Mediterranean, leaving corruption and infection wherever they happened to stand. These statements, though doubtless exaggerated, were believed by the people, and in what spirit they were met will be seen. Some

*Op cit., p. 28.

committed suicide in their frenzy. Many villages and towns, forsaken by the terrified inhabitants, and desolated by the plague, were left entirely empty and silent as the house of death. Rich men and merchants, in the hope of diverting the wrath of Heaven, carried all their money and valuables to the monasteries, when the monks, fearing the infection, closed their gates against both them and their treasures. By reason of the churchyards being full, in Avignon, the Pope was under the necessity of consecrating the water of the river Rhone as a burial-place—giving them at least the advantages of holy water, if not consecrated earth.† In Padua, when the plague disappeared, two-thirds of its inhabitants had also disappeared, most having fallen victims. The Venetians fled to the islands, forsaking the city and losing three out of every four. The dead were carried out of towns to be buried in pits, and the populace fancied that, from indecent haste, many were thrown in alive. Italy was reported to have lost one-half of its inhabitants. Churches were shunned as places of infection, and enriched, at the same time, with many donations and bequests. So many sudden deaths had engendered endless disputes as respects inheritance; and, when the disease passed away, men were amazed to observe how great was the proportion of lawyers to the rest of the community.

To show how the superstitions affected all classes, it is only necessary to mention, that by the most skilful physicians the black plague was ascribed to the grand conjunction of Saturn, Mars and Jupiter in the sign of Aquarius, in the year 1345. Such conjunctions always foreboded horrors to men, and every plague was in some way connected with the stars.

Such was the black death, which, towards the close of the year 1348, entered England, and committed unprecedented ravages, leaving, according to the extravagant calculations of Anthony Wood, scarcely a tenth part of its population; and the bodies of many, who were not affected by the pestilence, were weakened by the influences which operated in sweeping away so many, and few minds escaped the terror and superstitious dread arising from such heavy calamities.

About the first of the morbid mental derangements which followed this terrible manifestation of physical disease, we perceive existing among the brotherhood of the Flagellants, a sect which first sprang up in Hungary, and subsequently in Germany. Men, women and children of all ranks entered this order. They marched about in procession, each Flagellant with a red cross on his back, breast and cap, and carrying a triple scourge, attracting attention by the pomp of tapers and superb banners of velvet and cloth of gold. These arrogated to themselves such influence, and swayed so completely, at first, the disturbed minds of the people, that the church became jealous of them, as interfering with its own power for evil, as they claimed the privilege of absolving themselves; and a reaction being created against them in the fickle-minded populace, they were put down by relentless rage and persecution.*

But upon none did popular rage, induced by delusion, fall so heavily as upon the unfortunate Jews. The persecution of the Jews in those days began at Chillon, and spread from Switzerland through Europe.

---

†Hecher,—"Epidemics of the Middle Ages," p. 26. Phil. edition, 1837.

*Op cit., p. 35, 36, *et seq.*

Pestilence was ascribed, in those days, to poisoned wells, and the wells were said to be poisoned by the Jews. Many of these poor Jews, put upon the rack, confessed all that was required of them, and told tales of bags of poison sent among the faithful of Israel by the great rabbi of Toledo. Wells were bricked over, and buckets removed. The Jews of Basle were shut up in a wooden building, and then smothered and burned alive. The same was the case at Freyburgh. At Spires, the Jews withdrew to their houses, and setting fire to them, burnt themselves and all they had with their own hands. At Strasburgh, two thousand Jews were burned alive on their own burial-ground; and those who, in frantic terror, broke their bonds and fled were pursued and murdered in the streets.

It was, then, among people weakened physically and mentally by these desperate affliction and emotions, that there arose certain dancing manias, forming a fresh disease, afflicting both body and mind.

The same generation that had seen the terrors of the black death saw, some twenty years afterwards, men and women dancing in a circle, shrieking, and calling wildly on St. John the Baptist, and at last, as if seized with an epileptic fit, tumbling upon the ground, where they desired to be trodden upon and kicked by the bystanders, who were ready to do it cheerfully. Others, like themselves, with diseased bodies and minds, became affected by sympathy, and that disease called St. John's dance, which was supposed to be a form of demoniacal possession, spread over the entire Netherlands. They exorcised and made wonderful confessions.—Their numbers increased so rapidly that they became, in their turn, objects of fear and dread, and communicated their morbid fancies—such as a furious hatred of the red color, with the bull's desire to tear red cloths and rags in pieces,—a detestation of pointed shoes, and other matters of fashion against which the priests had declaimed from their pulpits. This class of persons became so numerous and violent as to intimidate the civil authorities, and in Liege an ordinance was issued to the effect that none should wear any but square-toed shoes. This epidemic mania appeared also at Metz and Cologne, and extended through most of the cities of the Rhine.*

The next epidemic, incident to these times, which we are called upon to notice was that which broke out at Strasburg, where the dancers were cared for by the town authorities, and conducted to the chapel of St. Vitus, a youthful saint martyred in the time of Diocletian. The first distinct account of this disease is to be found in the writings of Plater and Senertus, both of whom lived about the close of the sixteenth century. The name, St. Vitus' dance, by which it is now familiarly known, was derived, according to Hirst, from the chapel of St. Vitus, near Ulm. Women laboring under a certain nervous affection were in the habit of resorting thither every spring, where they danced violently and unremittingly from morning till night, until, in short, they were completely exhausted, or fell down into a kind of swoon or extacy, such as we sometimes see, in times of violent religious excitement at camp meetings at the present day. By this means they fancied themselves cured for one year. The disease was thought to arise from the malicious doings of Satan, and was generally treated by exorcism.

---

*Hecher on the Dancing Mania, p. 15.

The monks of the convent of St. Korbcy were said to be particularly fortunate in casting out the evil spirit, through the divine influence of St. Veit, their patron saint. A legend suited to the peculiar emergency could be made out in favor of this saint, because little was known concerning him; and, therefore, he, and he alone, was competent to cure the dancing plague. But the plague spread notwithstanding, and the physicians, quite as benighted as the subjects of the disease, regarding it as a peculiarly spiritual question, left it entirely to the care of the church; and for a century women went annually to the chapel of St. Vitus, on St. Vitus' day, to dace away the fever that had accumulated during the previous twelve months. Such were some of the characteristics of what was termed St. Vitus' dance. At first it attacked people of all ranks, but more especially those who led sedentary lives, and compelled them to dance, even to death—sometimes to dash out their brains against a wall, or to drown themselves.

The next dancing mania we are called upon to notice was that which arose in Apulia, among those who had been, or fancied they had been, bitten by a ground spider called tarantula. At the close of the fifteenth century the fear of this malady had spread beyond Apulia. Those who were bitten were said to have become melancholy, very open to the influence of music, given to fits of weeping, wild joy, and fits of dancing, longings, and fatal paroxysms either of laughter or sobs.

It was believed that the poison of the tarantula could only be worked off by those in whom it begat a violent energy of dancing; it passed out then with the perspiration; but if any lingered in the blood, the disorder became chronic or intermittent, and the afflicted person would be liable to suffering and melancholy, which whenever it reached a certain length, would be relieved by dancing.

The tarantali, or persons bitten by the tarantula, had various whims, violent antipathies to, and preferences for colors: most of them were wild in love of red; many were excited by new objects, &c. They were unable to dance except to music, and were under the necessity of having certain tunes called tarantellas performed; and the tarantella which suited one would not suit another, for some required a lively measure, others a melancholy one; some needed a suggestion of green fields, in both music and words, which must accompany. Nearly all required some reference to water, were mad in longing for the sea, and would be extatic at the sight of water in a pan. Some, after dancing, would plunge their heads into a tub of water. Some would dance with a cup of water in their hands.

In the beginning of the seventeenth century the cure of the tarantali was attempted on a large scale. Women, always foremost in acts of benevolence, employed bands of musicians to go about from village to village playing tarantellas for the benefit and relief of the afflicted, and the period of tarantella playing was called the "Woman's Carnival." The good creatures did not scruple to neglect their honorable duties, and allow their families to suffer, in order to give their money to pay for the dances; and some spent large fortunes in furthering this epidemic delusion, supposing themselves engaged in a work of heaven-born charity. These carnivals often influenced the thoughts of hysterical women, who, at their approach, sickened, and danced, and for the time being supposed themselves well; the taranturi included, however, quite as many men as women.

We cannot account for these moral epidemics of the middle ages in any more satisfactory way than by supposing them the result of physical debility, brought about by accumulated and prolonged bodily suffering, and in some way connected with the physical epidemics which committed such ravages about the same time, or a little previous.

It is a singular fact, recorded in connection with these epidemics, showing how mysterious are all the laws of nature, that the decrease of population, which one would suppose to result from such visitation as the black death, was in a great measure obviated by the extraordinary fruitfulness of marriage;* and it is highly probable that the unstrung nerves and physical weakness transmitted to children by unhealthy parents—such as had suffered by the influence of these calamities—had some influence in the development of the moral epidemics that followed and have been alluded to. When bodies are ill clad, ill nourished, ill housed, or by late sickness or other causes depressed, minds are apt to suffer in a corresponding manner. Any one who has experienced a severe fit of illness, which has produced great physical debility, is well aware to what extent the mind suffers, until the natural strength of the body is completely restored. The examples given show how bodies thus debilitated contained or transmitted minds equally debilitated and unhealthy, and peculiarly prone to be led away by the first lunatic fancy which presented itself; and therefore people abounded who were ready to believe themselves transformed into wolves and other wild beasts,—that they were witches, or possessed of evil spirits, &c. It is said that even the most sceptical could not shake off the influence of the popular credulity. A certain bishop of Foligno suffered himself, in bravado, to be bit by a tarantula; but even he, to become cured, had to throw aside his episcopal dignity, and take to dancing.

In 1556, a number of children, brought up in the city of Amsterdam—girls as well as boys—to the number of sixty or seventy, were attacked with an extraordinary disease. They climbed like cats on the walls and roofs. Their aspect was alarming, they spoke foreign languages, said wonderful things, and even gave an account of all that was passing in the municipal council. They ran in groups of ten or twelve through the public squares, went to the rector, and reproached him with his most secret actions. It is also asserted that they discovered several plots against the Protestants; and the faculty of prophesying, foretelling the future, and speaking in foreign languages appeared really to exist in this epidemic.†

But it is unnecessary to go back to the dark ages for illustrations, as such occur in our own times, and under the immediate observation of all persons. The delusion of "Millerism," which caused so much insanity, is well known to all who take any interest in the chronicles of human action; therefore it is only necessary to glance at it in this connection. Another, something similar, occurred about the same time in Sweden. The disease was characterized by two striking and remarkable symptoms; the one *physical* consisted of a spasmodical attack, involuntary contraction, contortions, &c.; the other *mental* was announced by an extacy, more or less involuntary, during which the patient believed that he saw

*Hecker.—"Treatise on the Black Death," p. 33.

†Van Dale.—"De l'Idolatriæ Praef," pp. 18 and 19.

and heard things divine and supernatural, and was instigated to speak, or, as the people expressed it, to preach. Many medical men considered this disease as one form of the chorea of the middle ages.

During these extacies the persons attacked were remarkable for an irresistible loquacity, a constant mania for preaching the Word of God, and for visions and prophesyings. In consequence of the peculiar tendency of this singular affection, it has been called *the preaching disease.* Most of the faculty who witnessed these paroxysms have compared them with somnambulism, or the magnetic sleep; but no one has been able to say positively that they belonged to either of these states. The sick persons frequently spoke of the visions they had had of heaven and hell, or angels, &c. They also predicted the end of the world, the last judgment, and the day of their own death—always with the assumption that their predictions were real and holy prophecies. It will be remembered that the greater number of the convulsed of St. Medard also predicted that the end of the world would occur on a day which they fixed; but, as with the Swedes and Millenarians, their prophesies were not accomplished.

These extatics, when the paroxysm was over, appeared as though they had emerged from a dream. They averred that they had seen supernatural sights, and recited the prophecies,—that they had seen the place of punishment of the condemned, and also the elect seated at the Lord's table. The greater number of the persons attacked were of the lower order. It was a physicial contagion, brought on by imitation. In one year, several thousand persons had the epidemic. A development of the intellectual faculties was not remarked in this disease; or, if it did exist, it was an exception. The greater number of the discourses and sermons were paltry and deviod of ideas, often consisting of pure nonsense, more frequently of exclamations repeated unto satiety, and continual repetitions of the same trifles, uttered in a sententious tone. Fanaticism, ignorance, and the thousands of religious tracts distributed amongst the people, had, according to the opinion of the Swedish faculty, induced a state of preparation for this epidemic.*

The influence of certain atmospheric conditions upon the mental as well as physical state of individuals has long been noticed, and there are few individuals who have not experienced this in their own persons. The influence of moist air upon the dispositions of the mind has long been known. It has also been noticed that certain atmospherical phenomena have preceded or attended suicidal epidemics; and whether certain electrical conditions of the atmosphere have or have not an influence in spreading the suicidal and other moral epidemics is a point not easily determined. That a certain degree of eccentricity, bordering on aberration, is, in certain individuals, in whom there lurks a latent predisposition to insanity, occasionally made manifest, is well known. "With our present amount of knowledge," says Dr. Winslow,† speaking of imitative or epidemic suicide, "of the subtle principle of contagion, it is difficult to say whether an effluvium may not be generated in such cases, which, un-

"*Memoire sur l'Extase religieuse epidemique," par M. le Docteur C. N. Souden.—Quoted from *Gazette Medicale* by Dr. D. Boismont, in his treatise on Hallucinations, p. 230, 231. Philadelphia edition, 1853.

†"Anatomy of Suicide," page 113.

der certain conditions of the system, may communicate disease. We cannot possibly say that such is not the case," says he, "though we are by no means willing to admit that the disposition to suicide may be propagated by contagion—using the term in its usual acceptation."

Whether suicide is or is not propagated by contagion, we cannot presume to decide; but that, in certain persons of a susceptible, nervous temperament, it is perpetrated by a morbid disposition to imitate—the same as hysteria, chorea, and other nervous affections—cannot be doubted. A man once hung himself on the threshold of one of the doors of the corridor at the *Hotel des Invalides.* For two years previous no suicide had occurred, but in the succeeding fortnight *five* invalids hung themselves on the same cross-bar, and the passage had to be closed. In one of the Berlin hospitals, some fifty years since, a young woman, of robust frame visited one of the patients. On entering the ward, she fell down in strong convulsions. Six female patients who saw her became at once convulsed in the same way, and, by degrees, *eight* others passed into the same condition for four months, during which time four nurses followed their example. They were all between sixteen and twenty-five years of age. Some years since, in one of our popular boarding-schools for young ladies, a pupil became affected by chorea. Her contortions being perceived by the school, this case was soon followed by another, and still another until the disease became regularly epidemic. A judicious physician being called in, proposed that cauterization by a red-hot iron should be applied to the next case which occurred: this prescription becoming generally known through the school, no more cases occurred.—In the olden time, the ladies of Miletus, in a fit of melancholy for the absence of their husbands and lovers, resolved to hang themselves, and, as in all fashionable amusements, vied with each other in the alacrity with which they carried on their work of self-destruction. Sydenham informs us that at Mansfield, in the month of June, suicide prevailed to an alarming degree, from causes wholly unknown. The same thing happened at Rouen, in 1806, at Stuttgart, in 1811, and at a village of St. Pierre Montjean, in the year 1813. One of the most marked suicidal epidemics was that which prevailed at Versailles, in the year 1793: in one year the number of suicides was thirteen hundred—a number entirely out of proportion to the population.

A suicidal epidemic prevailed in the New York State Lunatic Asylum, in July, 1851, and is alluded to by Dr. Benedict in his report for that year. Out of four hundred and sixteen patients, at that time in the institution, the suicidal propensity existed in sixty-six. The first successful attempt was made on the 12th of July, by a female of the most intelligent class. Her melancholy end became known to her companions, with whom she was a favorite, and on the following day two others in the same hall were overheard devising a plan for their own death. The large number of forty-four patients were admitted during the month of July, nineteen of whom were suicidal. Two patients, who had long been in the house, and never manifested suicidal propensities, attempted it during this month, *though they had no knowledge of what had occurred in another part of the building.* A female attendant took, on the same day the above attempt was made, an ounce of laudanum, "because she liked it." On the 17th a patient, believed to be entirely ignorant of what had happened previously, attempted strangulation, and continued to repeat the attempt, until restrained by mechanical means. On the 20th a patient tried to open a vein in the neck, and on the 22d another who knew of the suicide, and was, no doubt, influenced by it, attempted her destruction. From the 14th to the end of the month fourteen attempts were made by eight different persons, and twelve others, in whom the propensity was strong, required careful and constant observation. The epidemic prevailed from the 12th to the end of July, after which time it gradually subsided, and left the minds of most of the patients. No suicidal attempt was made in the month of August in any part of the house.*

The young women of Marseilles were at one period seized with a propensity to commit suicide. A law was passed to the effect that the body of every female who committed self-murder should be publicly exposed after death. The beneficial effect was apparent immediately. No more suicides occurred: the sense of outraged modesty and shame overcame at once this morbid propensity to self-destruction.

That the *primary* cause of many of these moral epidemics was some physical disturbance, or derangement of bodily function, there can be little doubt.

"The origin of self-destruction," says Dr. Winslow,† "is more frequently dependent upon derangement of the *primæ viæ* than is generally imagined."

The great influence of indigestion in causing mental disquietude has been noticed in a previous article, and that it has been felt by many persons in power whose bowels were constipated, is well known. Lord Byron says, in one of his letters, that nothing rendered his intellect so clear and vigorous as a dose of Epsom salts. Robespierre, the bloodthirsty tyrant of the French Revolution, was said to have had some derangement of the liver, and was habitually constipated. After death his bowels were found one adherent mass. It would be interesting to investigate the physical causes which operated in determining the character of this bloodthirsty, diabolical monster.

Coleridge, in his "Biographia Literaria," attributes much of the irritability of literary men to derangement of the healthy state of the physical frame. The late Dr. Brigham furnished an illustration of this state of things in his own person, and in his writings he also alludes to it.—Naturally one of the kindest and most benevolent of men, he also possessed much nervous irritability. He was, to use a common phrase, "quick tempered." This, to those who did not understand him, was sometimes very disagreeable, and called forth much forbearance on the part of his friends, and self-control on the part of himself. Much of this was attributable to his state of health, and the nervous irritability incident to excessive physical and mental labor, and its influence on a delicate organization.

The poet Pope had disease of the stomach and liver, producing hypochondriasis; and much of his malignity, petulance and personality was dependent, no doubt, upon this. Yet Bolingbroke observed to his friends that he "had known him for thirty years, and that he was the kindest

---

* See Report of Dr. Benedict to the Managers of the New York State Lunatic Asylum, for 1851.

† "Anatomy of Suicide," p. 195.

hearted man in the world." The following account of the poet is given by Dr. Johnson: "Pope's constitution," says he, "which was originally feeble, became so debilitated that he stood in perpetual need of female attendance; and so great was his sensibility to cold, that he wore a fur doublet under a shirt of very coarse, warm linen. When he arose he invested himself in a bodice, made of stiff canvas, being scarcely able to hold himself erect till it was laced, and he then put on a flannel waistcoat. His legs were so slender that he enlarged their bulk with three pairs of stockings, which were drawn on and off by the maid, for he was not able to dress or undress himself, and he neither went to bed nor rose without help." "His frequent attacks of indigestion made him, at times, a perfect picture of wretchedness and misery. It clothed everything with a gloomy aspect, made him quarrel with his friends and domestics, and he has been known to say that he sighed for death as a reprieve from bodily and mental agony. Sir Samuel Garth was frequently consulted when he had these attacks; and it was only by exacting strict attention to diet, and exhibiting medicines, that he was enabled to restore the mind of the poet to a healthy tone." This physical ailment, as it often does when long continued, ultimately affected the cerebral functions. At times he had symptoms of pressure on the brain, or, at least, of an unequal and imperfect distribution of blood to that organ.*

"There are crimes," says D'Israeli, "for which men are hanged, but of which they might easily have been cured by physical means." It is hard for the world to believe such doctrines, particularly if applied to those homicides who have committed great and revolting crimes, whether in a paroxysm of epileptic mania or laboring under the influence of a delusion which controlled, more or less, all the mental operations. Disease of the stomach frequently excites the suicidal disposition. Hepatic affections are almost always accompanied by depressions. Derangement of the uterine functions is also frequently the cause of great mental depression.

Some German writers lay great stress on the connection of insanity, particularly suicidal insanity, with derangement of the cutaneous secretions; but as we propose to recur to these sympathies again, in treating of the influence of the mental faculties of disease of individual organs, we merely glance at them at present.—*American Journal of Insanity.*

*"Anatomy of Suicide," by Forbes Winslow, p. 198.

